# The Biological Effects of Sex Hormones on Rabbit Articular Chondrocytes from Different Genders

**DOI:** 10.1155/2014/932737

**Published:** 2014-06-03

**Authors:** Shwu Jen Chang, Shyh Ming Kuo, Yen Ting Lin, Shan-Wei Yang

**Affiliations:** ^1^Department of Biomedical Engineering, I-Shou University, Kaohsiung 81346, Taiwan; ^2^Department of Orthopedics, Kaohsiung Veterans General Hospital, 386 Ta-Chung 1st Road, Kaohsiung 81300, Taiwan

## Abstract

The aim of this study was to investigate the biological effects of sex hormones (17**β**-estradiol and testosterone) on rabbit articular chondrocytes from different genders. We cultured primary rabbit articular chondrocytes from both genders with varying concentration of sex hormones. We evaluate cell proliferation and biochemical functions by MTT and GAG assay. The chondrocyte function and phenotypes were analyzed by mRNA level using RT-PCR. Immunocytochemical staining was also used to evaluate the generation of collagen-II. This study demonstrated that 17**β**-estradiol had greater positive regulation on the biological function and gene expressions of articular chondrocytes than testosterone, with the optimal concentrations of 10^−6^ and 10^−7^ M, particularly for female chondrocytes.

## 1. Introduction

Osteoarthritis (OA) is a degenerative joint disease that causes cartilage erosion, synovial inflammation, and osteophyte formation. Furthermore, articular cartilage has a limited self-healing potential after injury or degeneration, and the healing and repair of injured cartilage remains a difficult clinical problem. The incidence of OA increases with age in both genders, but there is a higher prevalence in females after the age of 50 years [1, 2]. Sex hormones, especially estrogens and androgens, have been considered to be possible factors for the predisposition to OA, and the depletion or altered metabolism of these hormones has been regarded as a risk for OA [3–5]. However, the correlation between sex hormones and OA is inconsistent. The effective doses on articular cartilage are inconclusive, and varying results have been reported. Ng et al. reported that physiological doses of estradiol did not alter proteoglycan synthesis for fish chondrocytes [6]. Other studies stated that supraphysiological estradiol protects articular chondrocytes against reactive oxygen species-induced damage, and antioxidative effects are observed after repeated physiological estradiol treatment [7]. Moreover, a decrease of collagen II degradation products in the urine has been observed following estrogen application in ovariectomized rats [8]. Claassen et al. showed a statistically significant decrease in the GAG content in miniature pigs with an experimentally induced estrogen deficiency in comparison with nontreated animals [9]. Furthermore, collagen II synthesis was found to be increased when chondrocytes from the rib growth zone of female rats were incubated with estradiol, supporting the hypothesis that estradiol deficiency in postmenopausal women might be associated with a higher incidence of OA [10]. In another study, Irie et al. reported that castration led to an increase in apoptosis and a decrease in the proliferation of chondrocytes in the growth plate; in addition, fewer chondrocytes were observed in the castrated rabbits than in normal animals of the same age [11].

To evaluate the effect of sex hormones (estradiol and testosterone) on the physiological regulation of chondrocytes, we designed experiments to investigate the biological effects of sex hormones on the rabbit chondrocytes from different genders. OA is characterized by the loss of collagens and proteoglycans in the extracellular matrix (ECM) of articular cartilage. Matrix metalloproteinases (MMPs) degrade several ECM molecules, including cartilage proteoglycan and collagen II. Tissue inhibitor of metalloproteinase (TIMP) is a glycoprotein that inhibits all MMPs on a 1 : 1 basis by forming high-affinity complexes. Some studies have suggested that there may be an overexpression of MMPs relative to the TIMPs in OA joints [12]. In addition to Col-I, Col-II, and aggrecan gene expression, we additionally addressed whether MMP-3 and TIMP-3 gene expression was affected differently in the genders. We cultured primary rabbit articular chondrocytes following incubations with sex hormones and used MTT assay, GAG assay, and real-time reverse transcription polymerase chain reactions (RT-PCR) to analyze the function and phenotype of the chondrocytes. Immunocytochemical staining was also performed at the end of culture.

## 2. Materials and Methods

### 2.1. Materials

*β*-Estradiol was purchased from Sigma (USA). Testosterone was purchased from Fluka (Switzerland). All of the other chemicals used in this study were of analytical reagent grade and were used without further purification.

### 2.2. Chondrocyte Isolation

Chondrocytes were isolated from the articular cartilage of six male and six female Japanese white rabbits (12 weeks of age). All procedures conformed to the guidelines of the Institute of Animal Care and Use Committee of I-Shou University. After aseptic preparation, the articular cartilage from the knee joint of the hind leg was dissected using a surgical blade and minced into 1 mm^3^ samples. These samples were rinsed twice with phosphate-buffered saline (PBS) and digested with protease (2 mg/mL, Sigma) for 2 hours followed by 0.2% collagenase II (Sigma) in PBS at 37°C for 3 h. The digested cell suspension was centrifuged at 1,500 rpm for 5 min to pellet the chondrocytes. The cell pellet was resuspended (in a 75 cm^2^ flask) in Ham's F12 medium (GIBCO) supplemented with 10% fetal bovine serum and antibiotics (200 U/mL penicillin/streptomycin), and the cells were cultured in an incubator at 37°C and 5% CO_2_.

### 2.3. Cell Culture and Sex Hormone Incubation

Primary rabbit articular chondrocytes from both genders were cultured separately. The second passage of chondrocytes was used, and the cells at a final density of 5 × 10^4^ cells/mL were incubated with various concentrations of sex hormones (10^−5^, 10^−6^, 10^−7^, 10^−8^, and 10^−9 ^M), either *β*-estradiol or testosterone, for 3 days. An untreated culture was used as the control.

### 2.4. MTT Assay

Cell viability was determined using MTT assay. After exposure to sex hormones for 3 days, MTT solution was added, and the cells were incubated for another 3 h. The supernatant was removed from the precipitated reaction products, and the precipitates were dissolved in dimethylsulfoxide (DMSO). The absorbance was measured using a multiplate reader (Thermo Scientific) at 570 nm.

### 2.5. GAG Assay

A commercially available GAG assay kit (Blyscan Assay Kit) was used to determine the total amount of GAG in the chondrocyte cultures. Briefly, culture medium samples were harvested and reacted with the Blyscan dye reagent that was composed of 1,9-dimethylmethylene blue (DMMB) for 30 min, and the unbound dye solution was removed by centrifugation. The bound dye was released from the insoluble GAG-dye complex using a dissociation reagent and quantified spectrophotometrically by measuring the absorbance at 650 nm. The total amount of GAG was determined using a standard curve that was generated using chondroitin 4-sulfate as a standard.

### 2.6. Real-Time PCR Analysis

The expression levels of collagen I (Col-I), collagen II (Col-II), aggrecan, TIMP-3, and MMP-3 were evaluated at the mRNA level using RT-PCR. Briefly, the total RNA was extracted using a Micro-to-Midi Total RNA Purification System according to the manufacturer's protocol. A total of 5 *μ*g of total RNA was used to synthesize cDNA using the SuperScript III First-Strand Synthesis System (Invitrogen). The cDNA was amplified using SYBR Green PCR reagents, and the RT-PCR reactions were conducted using the StepOne Real-Time PCR System (Applied Biosystems). An initial activation step was performed at 95°C for 30 sec, followed by 40 cycles at 95°C for 3 sec and 60°C for 30 sec. All of the reactions were performed in duplicate, followed by a melting-curve analysis for each RT-PCR run. The expression levels were calculated using the comparative CT method [13]. The following primer sequences were used: aggrecan, 5′-ATCTACCGCTGTGAGGTGAT-3′ (forward) and 5′-CTCCTGGAAGGTGAACTTCT-3′ (reverse); collagen type I, 5′-ACGCATGAAGGCAAGTTGGGTAG-3′ (forward) and 5′-AGCAGTGGTTACTACTGGATCGATC-3′ (reverse); collagen type II, 5′-AAGAGCGGTGACTACTGGAT-3′ (forward) and 5′-ACGCTGTTCTTGCAGTGGTA-3′ (reverse); TIMP-3, 5′-TCTGCAACTCCGACATCGTG-3′ (forward) and 5′-CGGATGCAGGCGTAGTGTT-3′ (reverse); MMP-3, 5′-GGCCATCTCTTCCTTCAG-3′ (forward) and 5′-GTCACTTTCTTTGCATTTGG-3′ (reverse); GAPDH, 5′-GTCAAGGCTGAGAACGGGAA-3′ (forward) and 5′-GCTTCACCACCTTCTTGATG-3′ (reverse). GAPDH was used as a housekeeping gene [14].

### 2.7. Immunocytochemistry of Cultured Articular Chondrocytes

For immunocytochemistry, cells were washed twice with PBS and fixed with 4% paraformaldehyde (Sigma) in PBS (pH 7.4) for 20 min at room temperature. After washing 3 times with PBS, the cells were permeabilized with 0.25% Triton X-100 (Sigma) in PBS for 20 min at room temperature and then blocked with 0.5% fetal bovine serum in PBS for 1 hour. The cells were then incubated overnight at 4°C with primary antibodies against Col-I (COL1A, sc-59772, 1 : 400) and Col-II (COL2A1, sc-52658, 1 : 500) (both from Santa Cruz Biotechnology, Santa Cruz, CA) [15]. The next day, the cells were washed 3 times with PBS and incubated with the corresponding secondary antibody in the dark for 1 h at room temperature; goat anti-mouse IgG-R (sc-2092, 1 : 400) was used for Col-I and goat anti-mouse IgG-FITC (sc-2010, 1 : 400) was used for Col-II (both from Santa Cruz Biotechnology). The cells were then stained with 4′,6-diamidino-2-phenylindole (DAPI; Invitrogen, USA) to visualize the nuclei. The stained cells were examined using a fluorescence microscope equipped with a digital camera (Olympus IX-71, Japan).

### 2.8. Statistics

All of the biochemical data are presented as the mean with 95% confidence intervals. The data between the different doses of sex hormones were analyzed using one-way independent ANOVA and post hoc Scheffe's tests. The data for the different hormones and genders were analyzed using multiway ANOVA. The statistical analysis was performed using the Statistical Package for the Social Sciences (SPSS version 10.0; SPSS, Chicago, IL). The chosen level of significance was *P* < 0.05.

## 3. Results and Discussion

### 3.1. MTT Assay and GAG Assay

**Figure 1:**
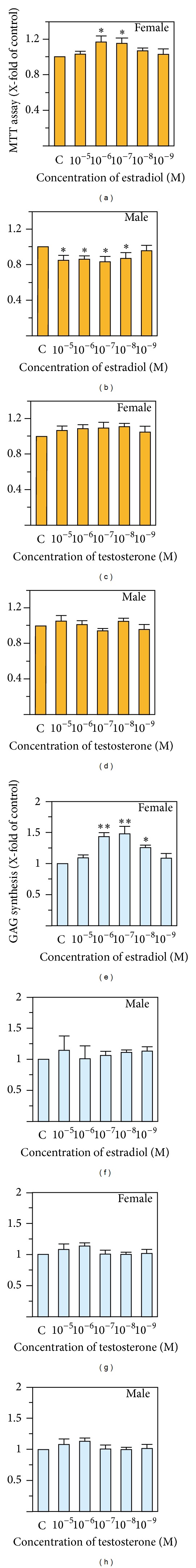
The results of MTT assays and GAG synthesis in articular chondrocytes from female and male rabbits following incubation with 17*β*-estradiol or testosterone for 3 days. The experiments were conducted in triplicate, and a mean ± standard deviation was expressed. Data were normalized to the control values, which were set at 1.0 (*P < 0.05, **P < 0.001).

A significant increase in cell proliferation, as indicated by the MTT assay, was observed in the chondrocytes from female rabbits following incubation with 10^−6^ and 10^−7^ M 17*β*-estradiol (*P* = 0.02 compared to the control) (Figure 1(a)). However, a significant decrease in cell proliferation was observed in male rabbit chondrocytes after incubation with 10^−5^ to 10^−8^ M (*P* = 0.02-0.03) 17*β*-estradiol (Figure 1(b)). No significant influence on MTT assay results was observed in chondrocytes incubated with testosterone, regardless of the origin of the cells (Figures 1(c) and 1(d)).

GAG synthesis was significantly increased in female rabbit chondrocytes incubated with 10^−6^ to 10^−8^ M 17*β*-estradiol, especially for 10^−6^ and 10^−7 ^M (*P* < 0.001) (Figure 1(e)). No significant effect on GAG synthesis was demonstrated in the male rabbit chondrocytes after any treatment or in the female ones after testosterone treatment (Figures 1(f), 1(g), and 1(h)).

### 3.2. Real-Time PCR Analysis

#### 3.2.1. Quantitative Evaluation of Col-I, Col-II, and Aggrecan mRNA Expression following Incubation with Estradiol

**Figure 2:**
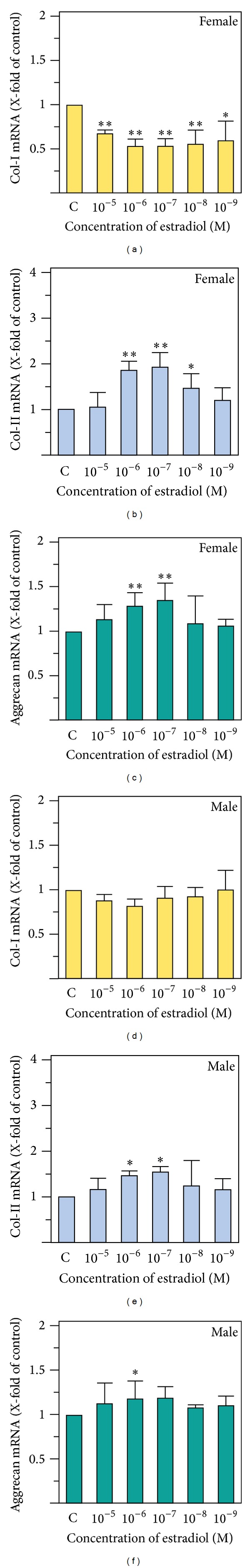
Influence of 17*β*-estradiol on the expression of collagen I (Col-I), collagen II (Col-II), and aggrecan mRNA in articular chondrocytes from female and male rabbits for 3-day culture. The experiments were conducted in triplicate, and a mean ± standard deviation was expressed. Data were normalized to the control values, which were set at 1.0 (*P < 0.05, **P < 0.001).

Compared to the control, an increase in expression of Col-II and aggrecan mRNAs was observed in female rabbit chondrocytes following incubation with 17*β*-estradiol, with particular statistical significance for 10^−6^ and 10^−7^ M (*P* < 0.001) (Figures 2(b) and 2(c)). Moreover, the female chondrocytes incubated with any dose of 17*β*-estradiol exhibited significantly suppressed expression of Col-I mRNA (*P* < 0.001) (Figure 2(a)). In the chondrocytes from male rabbits, aggrecan mRNA expression increased, but this increase was only statistically significant for 10^−6^ M (*P* = 0.04) (Figure 2(f)). No effect on the expression of Col-I mRNA was observed after incubation with 17*β*-estradiol (Figure 2(d)). The expression of Col-II mRNA increased significantly following incubation with 10^−6^ and 10^−7^ M (*P* = 0.02) 17*β*-estradiol (Figure 2(e)). The highest average increases in the expression of Col-II and aggrecan mRNAs were both detected in the chondrocytes from female rabbits incubated with 10^−7^ M 17*β*-estradiol, and the greatest average decrease in the expression of Col-I mRNA was detected in the chondrocytes from female rabbits incubated with 10^−6^ M 17*β*-estradiol (Table 1).

#### 3.2.2. Quantitative Evaluation of Col-I, Col-II, and Aggrecan mRNA Expression following Incubation with Testosterone

**Figure 3:**
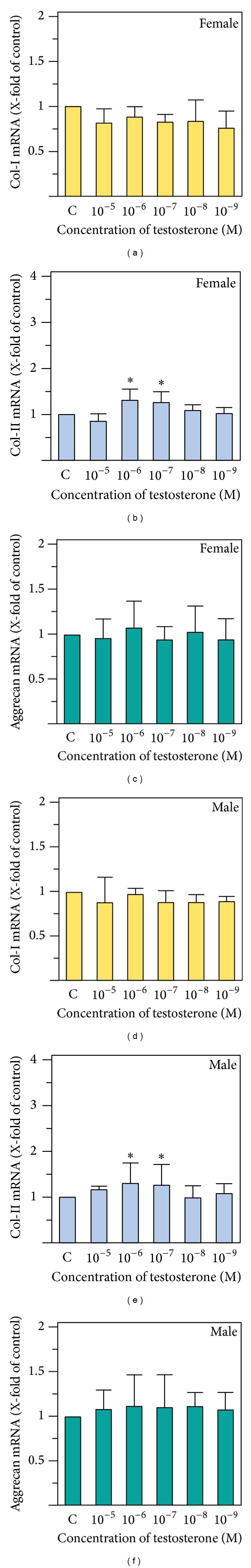
Influence of testosterone on the expression of collagen I (Col-I), collagen II (Col-II), and aggrecan mRNA in articular chondrocytes from female and male rabbits for 3-day culture. The experiments were conducted in triplicate, and a mean ± standard deviation was expressed. Data were normalized to the control values, which were set at 1.0 (*P < 0.05).

**Figure 4:**
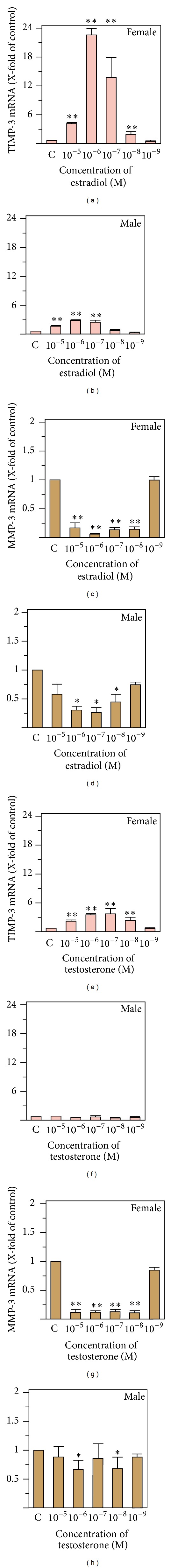
Influence of 17*β*-estradiol and testosterone on the expression of tissue inhibitor of matrix metalloproteinase-3 (TIMP-3) and matrix metalloproteinase-3 (MMP-3) mRNAs in articular chondrocytes from female and male rabbits for 3-day culture. The experiments were conducted in triplicate, and a mean ± standard deviation was expressed. Data were normalized to the control values, which were set at 1.0 (*P < 0.05, **P < 0.001).

In comparison with the control, increased Col-II mRNA expression was observed in the male rabbit chondrocytes after incubation with testosterone. Furthermore, significant effects were observed upon incubation with 10^−6^ and 10^−7^ M testosterone in chondrocytes from either male (*P* = 0.04 and 0.02) or female (both *P* = 0.02) rabbits (Figures 3(b) and 4(e)). Incubation with different doses of testosterone had no effect on the mRNA expression of Col-I or aggrecan in the chondrocytes from either gender (Figures 3(a), 3(c), 3(d), and 3(f)).

#### 3.2.3. Quantitative Analysis of TIMP-3 and MMP-3 mRNA Expression following Incubation with Estradiol

TIMP-3 mRNA expression was significantly increased in chondrocytes from female rabbits following incubation with 10^−5^ to 10^−8^ M (*P* < 0.001) 17*β*-estradiol and from male rabbits at concentrations of 10^−5^ to 10^−7^ M (*P* < 0.001) (Figures 4(a) and 4(b)). Furthermore, the expression of MMP-3 mRNA was significantly suppressed in the chondrocytes from female rabbits following incubation with 10^−5^ to 10^−8^ M (*P* < 0.001) 17*β*-estradiol and from male rabbits at doses of 10^−6^ and 10^−7^ M (*P* < 0.05) (Figures 4(c) and 4(d)). The greatest increase in the expression of TIMP-3 mRNA and decrease in the expression of MMP-3 mRNA were both detected in the chondrocytes from female rabbits incubated with 10^−6^ M 17*β*-estradiol (Table 2).

#### 3.2.4. Quantitative Evaluation of TIMP-3 and MMP-3 mRNA Expression following Incubation with Testosterone

Compared to the control, the expression of TIMP-3 mRNA was increased and the expression of MMP-3 mRNA was suppressed significantly in female rabbit chondrocytes after incubation with 10^−5^ to 10^−8^ M testosterone (*P* < 0.001) (Figures 4(e) and 4(g)). In the chondrocytes from male rabbits, incubation with testosterone had no significant influence on the expression of TIMP-3 mRNA (Figure 4(f)). However, a trend toward decreased MMP-3 mRNA expression was observed in male rabbit chondrocytes following incubation with testosterone, but this difference was only significant at the concentrations of 10^−6^ and 10^−8^ M (*P* = 0.03) (Figure 4(h)).

#### 3.2.5. Immunocytochemical Staining

**Figure 5:**
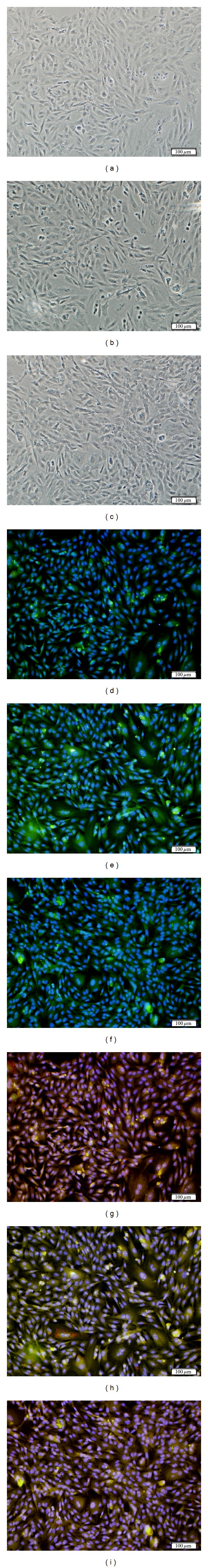
Immunocytochemical staining in female rabbit chondrocytes following incubation with sex hormones for 3 days. Optic microscopic view of (a) controls, (b) cells incubated with 10^−6^ M 17*β*-estradiol, and (c) cells incubated with 10^−6^ M testosterone. Immunocytochemical detection of Col-II of (d) controls, (e) cells incubated with 10^−6^ M 17*β*-estradiol, and (f) cells incubated with 10^−6^ M testosterone and immunocytochemical staining for combination of Col-I and Col-II in (g) controls, (h) cells incubated with 10^−6^ M 17*β*-estradiol, and (i) cells incubated with 10^−6^ M testosterone. All showed positive immunoreactivity for Col-II, and stronger positive staining was observed in the estradiol group.

Positive reactions with antibodies against Col-II were observed in groups of control, cells incubated with 10^−6^ M 17*β*-estradiol or testosterone. However, greater staining for collagen II expression was presented in the chondrocytes incubated with 10^−6^ M 17*β*-estradiol than in control cells or cells incubated with testosterone (Figure 5).

In our MTT assays, all doses (10^−5^ to 10^−9^ M) of 17*β*-estradiol resulted in increased proliferation in the female rabbit chondrocytes, although significant increases were found only at the doses of 10^−6^ and 10^−7^ M. In addition, a significant increase in the expression of aggrecan mRNA was observed at doses of 10^−6^ and 10^−7^ M 17*β*-estradiol (Figure 2(c)). These results can explain why female rabbit chondrocytes stimulated with 10^−6^ and 10^−7^ M 17*β*-estradiol exhibited the highest levels of GAG synthesis (Figure 1(e)). However, supraphysiological doses of 17*β*-estradiol (10^−5^ to 10^−8^ M) led to significantly lower proliferation in the male rabbit chondrocytes, whereas the physiological dose (10^−9^ M) had no effect. In contrast, no significant influence was observed based on the results of cell proliferation or GAG analysis after incubation with testosterone either in the female or in the male chondrocytes. Furthermore, no significant influence of testosterone on aggrecan gene expression was observed in both genders (Figures 3(c) and 3(f)), which corresponded to the GAG assay result (Figures 1(g) and 1(h)).

In OA cartilage, Col-II manifested a decreased staining and Col-I was found as a thin layer covering the surface of fibrillated cartilage [16]. Positive effects on the articular chondrocytes, including the upregulation of Col-II and aggrecan or downregulation of Col-I expression, were observed following incubation with 17*β*-estradiol in female chondrocytes. In contrast, an increase in only Col-II mRNA expression was observed at 10^−6^ and 10^−7^ M of testosterone in both genders (Figures 3(b) and 3(e)), with no significant difference between the genders (Table 1). In the comparison of different genders and sex hormones, the stimulation of Col-II mRNA expression and suppression of Col-I mRNA expression were significantly greater in the female cells with estradiol than in the male cells with testosterone (Table 2). In addition to Col-I, Col-II, and aggrecan, MMPs and TIMPs also play important roles in OA. MMPs degrade ECM molecules, including cartilage proteoglycan and collagen II, whereas TIMPs inhibit MMPs by forming high-affinity complexes. Several studies have demonstrated that, in the pathogenesis of OA, MMPs may be overexpressed relative to TIMPs [17, 18]. The increased expression of MMPs will degrade Col-II and aggrecan, whereas the expression of TIMPs will prevent the degradation of articular cartilage. In our experiments, the highest levels of TIMP-3 mRNA expression were observed in female chondrocytes after incubation with 10^−6^ and 10^−7^ M estradiol, and the lowest expression of MMP-3 mRNA was observed in female chondrocytes after incubation with 10^−6^ M estradiol. These results likely reflect the fact that GAG synthesis was the highest in the female chondrocytes at 10^−6^ and 10^−7^ M estradiol. Song et al. demonstrated that increased MMP-3 mRNA in the knee cartilage of ovariectomized rabbits could be suppressed by estrogen supplementation [19]. Similarly, our results indicated that supraphysiological doses (10^−5^ to 10^−8^ M) of 17*β*-estradiol could significantly suppress the expression of MMP-3 mRNA. Claassen et al. also showed significant suppressing effect of incubation with 10^−5^ M of 17*β*-estradiol on MMPs mRNA expression in female chondrocytes [20]. The gene expression results indicated that 17*β*-estradiol affected Col-I and Col-II in female rabbit chondrocytes, significantly promoting the expression of Col-II mRNA and suppressing the expression of Col-I. The concentrations of 10^−6^ and 10^−7^ M induced the most balanced collagen expression in articular cartilage. Furthermore, incubation of the female rabbit chondrocytes with 10^−6^ M 17*β*-estradiol produced the highest expression of TIMP-3 mRNA and the lowest expression of MMP-3 mRNA between the different sex hormones and genders.

According to RT-PCR, MTT, and GAG synthesis assays using female rabbit chondrocytes incubated with 17*β*-estradiol, the ideal concentrations for a positive effect on cartilage were 10^−6^ and 10^−7^ M, which are supraphysiological doses. However, the positive effect was reduced at a dose of 10^−5^ M versus 10^−6^ and 10^−7^ M. The higher concentrations are potentially toxic to chondrocytes. Previous studies demonstrated that 17*β*-estradiol applied at 10^−4^ to 10^−5^ M caused a significant loss of GAG in the fibrocartilage of the pubic symphysis in ovariectomized rabbits [21, 22]. No significant effect was observed for the physiological dose of estradiol in our studies. Claassen et al. also reported that incubation with physiological dose of estradiol alone did not significantly influence proline incorporation or collagen II synthesis in their study of cow chondrocytes [23].

Col-II is the main structural component of the ECM of articular cartilage. Col-I is not expressed in healthy articular cartilage, but it can be detected in degenerated cartilage or fibrocartilage. In our study, when chondrocytes were cultured in two-dimensional culture, their tendency to dedifferentiate was accompanied by a switch from collagen II to collagen I synthesis. Both Col-I and Col-II were observed via immunocytochemical staining, and a stronger immunoreaction for Col-II was observed in 10^−6^ M 17*β*-estradiol-treated cells than in testosterone-treated or control cells. The immunocytochemistry findings were consistent with a collagen-stimulating effect for sex hormones, which both stimulated Col-II mRNA expression and increased the volume of Col-II in the ECM. A stronger regulation was observed for 17*β*-estradiol than for testosterone.

## 4. Conclusions

The findings of our study suggest that 17*β*-estradiol plays a more important role than testosterone in the biological functions and gene expression profiles of chondrocytes, especially in female chondrocytes. Furthermore, both protective and stimulating effects of 17*β*-estradiol on female chondrocytes were observed with the greatest effects at 10^−6^ and 10^−7^ M in two-dimensional culture. Therefore, further studies are needed to test the effect of 10^−6^ or 10^−7^ M 17*β*-estradiol in chondrocytes using three-dimensional culture or* in vivo* animal studies.

## Figures and Tables

**Table 1:** The expression of collagen I and collagen II on different groups with analysis by RT-PCR.

Sex hormone	Expression of collagen I (X-fold of control)		Expression of collagen II (X-fold of control)	
	Male	Female	Male	Female
Estradiol				
10^−6 ^(*n* = 9)	0.77 (0.65 to 0.89)	0.52 *(0.41 to 0.64)	1.48 (1.39 to 1.56)	1.89 *(1.76 to 2.02)
10^−7 ^(*n* = 9)	0.89 (0.68 to 1.14)	0.56 *(0.47 to 0.65)	1.52 (1.39 to 1.65)	1.96 *(1.72 to 2.21)
Testosterone				
10^−6 ^(*n* = 9)	0.98 (0.87 to 1.09)	0.89 (0.78 to 1.01)	1.24 (0.77 to 1.71)	1.31 (1.09 to 1.53)
10^−7 ^(*n* = 9)	0.89 (0.68 to 1.10)	0.78 (0.66 to 0.91)	1.21 (0.74 to 1.68)	1.28 (1.05 to 1.51)

Values are the mean (95% confidence interval [CI]).

*n*: number of chondrocytes samples.

*P < 0.05 by multiway ANOVA.

**Table 2:** The expression of TIMP-3 and MMP-3 on different groups with analysis by RT-PCR.

Sex hormone	Expression of TIMP-3 (X-fold of control)		Expression of MMP-3 (X-fold of control)	
	Male	Female	Male	Female
Estradiol				
10^−6 ^(*n* = 9)	2.89 (2.68 to 3.11)	22.41 **(20.99 to 23.83)	0.31 (0.23 to 0.39)	0.09 *(0.07 to 0.11)
10^−7 ^(*n* = 9)	2.77 (2.62 to 2.92)	13.68 **(9.54 to 17.42)	0.24 (0.17 to 0.31)	0.18 (0.13 to 0.23)
Testosterone				
10^−6 ^(*n* = 9)	0.72 (0.69 to 0.75)	3.21 (3.11 to 3.31)	0.68 (0.56 to 0.81)	0.17 (0.15 to 0.19)
10^−7 ^(*n* = 9)	0.89 (0.78 to 1.01)	3.31 (1.72 to 4.91)	0.77 (0.50 to 1.04)	0.18 (0.14 to 0.22)

Values are the mean (95% confidence interval [CI]).

*n*: number of chondrocytes samples.

*P = 0.001 and **P < 0.001 by multiway ANOVA.
